# Bilateral deep transcranial magnetic stimulation of motor and prefrontal cortices in Parkinson’s disease: a comprehensive review

**DOI:** 10.3389/fnhum.2023.1336027

**Published:** 2024-01-24

**Authors:** Colleen A. Hanlon, Daniel H. Lench, Gaby Pell, Yiftach Roth, Abraham Zangen, Aron Tendler

**Affiliations:** ^1^Department of Cancer Biology, Wake Forest School of Medicine, Winston-Salem, NC, United States; ^2^BrainsWay Ltd., Jerusalem, Israel; ^3^Department of Neurology, Medical University of South Carolina, Charleston, SC, United States; ^4^Department of Life Sciences, Ben Gurion University of the Negev, Beersheba, Israel

**Keywords:** rTMS, Deep TMS, Parkinson’s disease, prefrontal cortex, neuromodulation

## Abstract

Parkinson’s disease (PD) is a prevalent neurodegenerative disorder characterized by both motor and non-motor symptoms, many of which are resistant to currently available treatments. Since the discovery that non-invasive transcranial magnetic stimulation (TMS) can cause dopamine release in PD patients, there has been growing interest in the use of TMS to fill existing gaps in the treatment continuum for PD. This review evaluates the safety and efficacy of a unique multifocal, bilateral Deep TMS protocol, which has been evaluated as a tool to address motor and non-motor symptoms of PD. Six published clinical trials have delivered a two-stage TMS protocol with an H-Coil targeting both the prefrontal cortex (PFC) and motor cortex (M1) bilaterally (220 PD patients in total; 108 from two randomized, sham-controlled studies; 112 from open label or registry studies). In all studies TMS was delivered to M1 bilaterally (Stage 1) and then to the PFC bilaterally (Stage 2) with approximately 900 pulses per stage. For Stage 1 (M1), two studies delivered 10 Hz at 90% motor threshold (MT) while four studies delivered 1 Hz at 110% MT. For Stage 2 (PFC), all studies delivered 10 Hz at 100% MT. The results suggest that this two-stage Deep TMS protocol is a safe, moderately effective treatment for motor symptoms of PD, and that severely impaired patients have the highest benefits. Deep TMS also improves mood symptoms and cognitive function in these patients. Further research is needed to establish optimal dosing and the long-term durability of treatment effects.

## Introduction

Parkinson’s disease (PD) is a progressive neurodegenerative disorder, second in frequency only to Alzheimer’s (GBD 2016 Parkinson’s Disease Collaborators, 2018). Traditionally, PD has been characterized as a motor system disorder with four prominent symptoms: bradykinesia, rigidity, postural instability, and tremor (Armstrong and Okun, 2020). There are also well-established non-motor symptoms including depression, apathy, sleep disorders, and a variety of autonomic symptoms. Among these non-motor symptoms, cognitive and mood effects are particularly pernicious as they can overshadow quality of life improvements provided by motor symptom treatments and increase caregiver burden (Benito-León et al., 2012; Lawson et al., 2016).

The clinical management of PD relies heavily on pharmacotherapy and may ultimately lead to invasive deep brain stimulation (DBS) for a subset of severe patients (Armstrong and Okun, 2020). First-line pharmacotherapeutic treatments generally treat the motor symptoms of PD very well, especially in the early stages of the disease process. Long-term use of many of the dopaminergic agents, however, can result in disabling side effects such as dyskinesias (Pringsheim et al., 2021). Furthermore, several symptoms such as freezing of gait, speech disturbances, apathy, and cognition are particularly resistant to pharmacotherapy (Vorovenci et al., 2016). While DBS or surgical ablation are alternatives available to many severe patients, these are invasive and not appropriate for all patients (Deuschl et al., 2022).

Transcranial magnetic stimulation (TMS) is a unique, non-invasive neuromodulation approach which may be able to fill this treatment gap. Through electromagnetic induction, single pulses of TMS can depolarize neurons in specific cortical areas of interest. When TMS is delivered in a repetitive manner over many sessions it can induce behavioral changes that endure beyond the length of the stimulation (review: Lefaucheur et al., 2020). Various forms of TMS are currently FDA-cleared for use in the treatment of multiple disorders of mood and arousal [e.g., major depressive disorder (Levkovitz et al., 2015), anxious depression (Pell et al., 2022), obsessive-compulsive disorder (OCD) (Carmi et al., 2019), and smoking addiction (Zangen et al., 2021)].

While TMS is not currently FDA-cleared for use in PD, there is a growing body of promising clinical research in this area (reviews: Cantello et al., 2002, Zhang et al., 2022). The majority of studies have applied TMS to a single cortical target – either the primary motor cortex (M1), supplementary motor cortex (SMA), or the prefrontal cortex (PFC). These studies have also largely used a flat figure-8 TMS coil, which can target a focal area in one hemisphere at a time. While some of these small studies have been promising, the effect sizes have been modest (Cantello et al., 2002). Given that PD contains both motor and non-motor symptoms, it may be valuable to stimulate the PFC as well as M1 in a treatment session. Additionally, as the behavioral manifestations of PD are typically bilateral, there has been emerging interest in the use of H-Coils (a specific type of TMS) as a tool to stimulate the left and right sides of the motor cortex simultaneously.

There have now been six published clinical trials that have delivered a two-stage TMS protocol with an H-Coil targeting both the PFC and motor cortex (M1) bilaterally. These H-Coils have been CE cleared for PD since 2013, but they are not yet FDA-cleared for this indication. This review summarizes the trial design and outcomes of these studies and provides perspective as to how this emerging protocol could fit into the continuum of clinical care for PD in the future.

## Methods

We performed a comprehensive PubMed search for clinical studies using Deep TMS™ studies for PD. Search terms included PD, Deep TMS, H-Coil, and rTMS. Results were limited to clinical trials and the English language.

**FIGURE 1:**
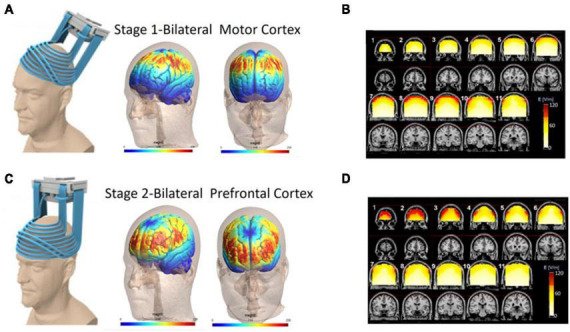
Electric field distribution for the H4 Coil at each location of the two-stage protocol. Six published clinical trials have delivered a two-stage TMS protocol with an H-Coil targeting both the prefrontal cortex and motor cortex bilaterally (220 PD patients total). In all studies, TMS was delivered to the motor cortex (Stage 1; **A,B**) and then to the prefrontal cortex (Stage 2; **C,D**) with approximately 900 pulses per stage. The electric field simulation is shown in two ways: cortical renderings (SIMNIBS software) of the field in the motor cortex position **(A)** and prefrontal cortex position **(C)**. Additionally electric field calculations from saline head models in these two configurations **(B,D)** are shown. For the saline models, the maps were adjusted to the average percentage of the maximal stimulator output required to achieve 120% of the hand rMT. The red pixels indicate field magnitude ≥ the threshold for neuronal activation, which was set to 100 V/m. Full field maps for the H2, H4, and H5 Coils (all of which are very similar) can be found in Supplementary material.

The search revealed six clinical trials which used a two-stage Deep TMS protocol involving PFC and M1 stimulation (Figure 1). The studies all used bilaterally symmetrical crown shaped H-Coils which had colocalized electric field distributions over the motor cortex (Supplementary Figure 1) and PFC (Supplementary Figure 2; Tendler et al., 2016). In this review, we focused on change in the total Unified Parkinson’s Disease Rating Scale (UPDRS) score (Goetz et al., 2008) and change in the motor subscale (Part III) of the UPDRS. These were the most frequently used end points and are a widely accepted scale for PD severity. Across the six studies, participants were evaluated in the same dopaminergic state, either ON or OFF their medications. Secondary endpoints were variable and included neuropsychological assessments and various objective measures of motor performance (e.g., timed up and go or finger/foot tapping).

The first published study was a prospective open label pilot study which led to CE clearance (Spagnolo et al., 2014). The second published study was a prospective randomized study wherein half of the patients received the two-stage protocol and the other half only motor cortex stimulation (Cohen et al., 2016). The third study was from an open label clinical registry evaluating the two-stage protocol with 30 day follow-up (Torres et al., 2015). The fourth study was sham-controlled and evaluated the effects of the treatment at 90 days (Cohen et al., 2018). The fifth study was a randomized, sham-controlled three arm study using a 30 day post treatment endpoint (Spagnolo et al., 2020). The sixth study was real world evidence from PD patients that were treated with the two-stage protocol while in an inpatient setting (Cont et al., 2022).

## Summary of the studies

### TMS protocol: all studies used a two-stage protocol

*Stage 1:* Resting motor threshold (MT) was measured on the more affected hemisphere (hemisphere contralateral to the predominantly affected side of the body). MTs were determined by finding the lowest stimulation intensity able to produce motor-evoked potentials of the abductor pollicis-brevis muscle in 50% of the pulses delivered. The coil was then placed over the motor cortex symmetrically and TMS was delivered at either 10 Hz (90% MT, Studies 1 and 5) or 1 Hz (110% MT, Studies 2 and 4). *Stage 2:* Immediately thereafter, the H-Coil was moved anteriorly along the midline 5.5–6 cm from the motor cortex wherein the peak electric field was located over the dorsolateral prefrontal cortex. TMS was delivered at 10 Hz (100% MT; 2 s train followed by a 20 s inter train interval, 42–45 trains, 840–900 total pulses). For each stage, 800–900 pulses were delivered for a total of 1,680–1,800 pulses per treatment day (Figure 1).

Tables 1, 2 contains a summary of the TMS parameters and primary results for motor and non-motor symptoms in each study.

**TABLE 1:** Overview of Deep TMS studies: designs and participant characteristics.

Study	References	Total sample size	Study design	Gender	Age	PD duration (years)	Motor (MDS)-UPDRS	Total (MDS)-UPDRS	Hoehn and Yahr	LEDD (mg)
1	Spagnolo et al., 2014	27	Open label	7F, 20M	60.1 ± 6.8	6.3 ± 2.8	39.6 ± 10.1	NR	2.2 ± 0.3	NA
2	Cohen et al., 2016	19	Open label (two active arms)	5F, 14M	60.9 ± 12.2	7.8 ± 6.5	Arm 1: 37 Arm 2: 26 (median)	Arm 1: 52 Arm 2: 41 (median)	2	416.3 ± 265.3
3	Torres et al., 2015	45	Chilean registry	19F, 26M	62.5 ± 1.6	9.8 ± 0.9	NR	70 ± 3.8	2.3 ± 0.1	470.72
4	Cohen et al., 2018	48	Sham controlled	16F, 32M	65.6 ± 7.5	5.1 ± 3.5	28.9 ± 8.9	41.6 ± 12.8	2	345.6 ± 290.2
5	Spagnolo et al., 2020	60	Sham controlled	18F, 41M	63.9 ± 10	7.6 ± 4.9	NR	42.4 ± 11.2	2	585.1 ± 304
6	Cont et al., 2022	21	German registry	8F, 13M	71.1 ± 11.4	NR	37.3 ± 10.9	NR	NR	NA

Mean ± SD displayed unless otherwise stated. NR, not reported; (MDS)-UPDRS, (Movement Disorders Society)-Unified Parkinson’s Disease Rating Scale; LEDD, Levodopa Equivalent Daily Dose.

**TABLE 2:** Summary of study parameters and primary results.

Study	References	Total sample size	Stage 1: M1 (Hz)	Stage 2: PFC (Hz)	Sessions	Change in motor symptoms	Change in non-motor symptoms	Other
1	Spagnolo et al., 2014	27	10	10	12	Yes, motor UPDRS *p* < 0.001	*	Effect largest in advanced disease
2	Cohen et al., 2016	19	1	10	12	Yes, motor UPDRS *p* < 0.04	*	Significant total UPDRS change, *p* < 0.02
3	Torres et al., 2015	45	1	10	12	Yes, motor UPDRS *p* < 0.0001	Yes, non-motor UPDRS *p* < 0.0001	Significant improvement remained at 30 day follow-up
4	Cohen et al., 2018	48	1	10	24	No significant difference between active/sham. Within the active there was significant improvement.	*	Primary endpoint was 90 days after treatment initiation. Effect was largest in advanced disease.
5	Spagnolo et al., 2020	60	10	10	12	Yes, total UPDRS worse side *p* < 0.04, better side *p* < 0.01	*	Significant improvement in UPDRS tremor subscale.
6	Cont et al., 2022	21	1	10	1–11	No. Trend toward UPDRS decrease (*p* = 0.1)	Yes, Becks Depression Inventory *p* = 0.015	Inpatient study. Large range of TMS sessions. Effects largest in advanced disease.

UPDRS, Unified Parkinson’s Disease Rating Scale. *Not explicitly assessed or reported.

### Deep TMS outcomes and efficacy

#### Study 1

Spagnolo et al. (2014) twenty-seven PD participants were administered 12 sessions of TMS over 4 weeks in this open label study. A two-stage protocol was used in which both M1 and PFC were stimulated at 10 Hz (840 pulses at each location; H2 Coil). The primary endpoint occurred immediately after the final treatment session. Deep TMS was performed while participants were ON their PD medications. UPDRS evaluations were performed while participants were OFF PD medications. As a group, the motor UPDRS III score significantly decreased by 27% from baseline to the end of treatment. A total of 81.5% of patients had a clinically meaningful reduction in the UPDRS III. This was positively correlated with severity, wherein more severely impaired patients had better odds of benefiting from Deep TMS.

#### Study 2

Cohen et al. (2016) nineteen participants with PD were administered 12 sessions of TMS over 4 weeks and randomized to either a two-stage protocol or M1 only stimulation. M1 stimulation was 1 Hz at 110% MT (900 pulses; H2 Coil). PFC stimulation was 10 Hz at 100% MT (900 pulses; H2 Coil). The primary endpoint occurred immediately after treatment and 30 days after treatment completion. Deep TMS was performed while participants were ON PD medications. In the two-stage protocol group, immediately after the last treatment session, there was a 15% reduction in the total UPDRS and 24% reduction in the motor UPDRS. The UPDRS score after TMS was not significantly different than baseline in the M1 only group. The two-stage protocol had a larger effect size than M1 only.

#### Study 3

Torres et al. (2015) forty-five PD participants received an average of 13.6 sessions of Deep TMS over 4 weeks in a clinical setting in this open label, registry study. M1 stimulation was 1 Hz at 110% MT (900 pulses; H2 Coil). PFC stimulation was 10 Hz at 100% MT (800 pulses; H2 Coil). The primary endpoint occurred at the end of treatment and 30 days after treatment completion. Clinical assessments were performed while participants were ON their PD medication. As a group, there was a 4.2-point improvement on the motor MDS-UPDRS after treatment, which was maintained after 30 days, and patients with greater severity fared better. There was improvement in all domains of the MDS-UPDRS III, as well as in assessments of gait speed, depression severity, balance, and autonomic symptoms. A 73% increase in daily ON time was reported.

#### Study 4

Cohen et al. (2018) forty-eight PD participants were administered 24 sessions of Deep TMS over 12 weeks (weeks 1–4: 3×/week; weeks 5–8: 2×/week; weeks 9–12: 1×/week) in this randomized, sham-controlled study. Forty-two of the participants from the Intent to Treat sample completed the full protocol. The reasons for drop out were not listed in the manuscript. Stimulation and clinical assessment of symptoms were performed ON PD medication. M1 stimulation was 1 Hz at 110% MT (900 pulses; H5 Coil). PFC stimulation was 10 Hz at 100% MT (900 pulses; H5 Coil). The primary endpoint occurred 90 days after treatment completion. The total and the motor UPDRS scores had improved between baseline and 90 days after treatment completion for both groups. There was a main effect of time but no significant main effect of treatment. Simple effects analysis revealed a significant decrease in UPDRS score over time in the active Deep TMS group (*F*_1_,_39_ = 8.6; *p* = 0.006), with no significant change in the sham group (*F*_1_,_39_ = 3.7; *p* = 0.06). In a secondary analysis of individuals classified as “responders” (UPDRS decrease of 4.5 points or more) and “non-responders” revealed that “responders” were older, used more levodopa and had a lower MT. Total UPDRS improvement correlated with severity and with disease duration. Patients with a motor UPDRS 25–50 had 40% response rate following active compared to 27% following sham treatment. Patients with disease >5 years had 50% response rate following active compared to 18% following sham treatment.

#### Study 5

Spagnolo et al. (2020) sixty participants with PD were administered 12 sessions over 4 weeks, randomized to 1 of 3 arms: two-stage protocol, motor cortex only, or sham study. Both M1 and PFC were stimulated at 10 Hz (840 pulses at each location; H5 Coil). Stimulation was performed ON PD medication except for the first and last session where participants were evaluated OFF their PD medications. The primary endpoint occurred 30 days after treatment completion. The group which received the active protocol had a 27% improvement in motor UPDRS score which was significantly greater improvement than sham (15%).

#### Study 6

Cont et al. (2022) twenty-one participants with non-idiopathic Parkinson’s syndromes. The patients were all required to have bradykinesia and at least one of the following features: rest tremor, muscular rigidity, or disturbances of posture and gait. They received up to 11 sessions in an inpatient setting for this open label study. M1 stimulation was 1 Hz at 90% MT (900 pulses; H5 Coil). The study demonstrated similar safety and efficacy for motor symptoms following Deep TMS. We included the study as it demonstrates the transdiagnostic relevance of Deep TMS for these motor symptoms observed in traditional PD as well as non-idiopathic PD. PFC stimulation was 10 Hz at 100% MT (800 pulses; H5 Coil). The primary endpoint was immediately after treatment completion. There was a lot of variation in the reported number of treatment sessions delivered (range 1–11), likely because patients were discharged from the hospital. Six of the 21 patients had post-treatment UPDRS. The authors conclude that the treatment significantly decreased the subjective main symptom severity. The authors stated that Deep TMS was particularly helpful for older subjects with motor symptoms and depression, including those with hypokinetic gait and freezing of gait. The consistency of these results (done in non-idiopathic PD) with Studies 1–5 highlight the potential transdiagnostic relevance of Deep TMS for motor symptoms frequently observed in traditional PD as well as non-idiopathic PD.

#### Deep TMS safety:

The number of patients with adverse events (AEs) is reported in Table 3. All AEs were minor and transient in their duration. This included AEs which are commonly reported across TMS studies (Tendler et al., 2023), such as headache and pain at the site of stimulation. Specifically, headaches, face pain and discomfort occurred in 16% of subjects. PD specific AEs included dizziness, nausea, sleepiness, and dyskinesias. Sleepiness occurred in 5% of subjects, dizziness and brief hypotension occurred in 3% of subjects, and transient dyskinesias in 3% of subjects (which abated within 15 min of TMS). The dropout rate for Deep TMS in PD studies was low (only 4 out of 220 subjects dropped out across six studies). The H2 and H5 Coils are almost identical to the H4 Coil, which has the lowest rates of AEs in a recently published compilation of AEs from five multicenter Deep TMS studies (Tendler et al., 2023).

**TABLE 3:** Adverse events.

Study	References	Head or face pain/discomfort	Dizziness	Nausea	Brief dyskinesia	Sleepiness	Other	Drop out
1	Spagnolo et al., 2014	1	2 brief hypotension	0	4	0	None	0
2	Cohen et al., 2016	9	0	0	0	3	1 visual hallucination	0
3	Torres et al., 2015	6	0	2	0	8	None	0
4	Cohen et al., 2018	5	4	2	0	0	4 pain, 2 general weakness, 2 gait disturbance	3
5	Spagnolo et al., 2020	3	1	0	3	0	None	0
6	Cont et al., 2022	12	0	3	0	1	1 pain, 1 tremor, 1 insomnia, gait drift	1

## Discussion

It has been over 20 years since Strafella et al. (2001) published the first body of work demonstrating that TMS to the PFC and to the motor cortex cause an increase in dopamine release in healthy controls and PD patients. Until recently however, we have not found a way to translate these brain imaging results into a therapeutic treatment for patients with PD. While most of the TMS studies to date have focused on stimulating either the motor cortex or the premotor cortex (often with low frequency TMS), the results have been mixed, possibly because the cumulative TMS dose in these studies has been too low or not broad and deep enough for these patients. In this manuscript we summarize a growing body of literature demonstrating the efficacy and tolerability of a unique, two-stage TMS protocol which stimulates both the PFC and the motor cortex bilaterally in PD patients using H-Coils – a specific type of TMS coil which has a relatively wide and deep electric field.

In aggregate, the studies in this review describe overall 220 patients that received this two-stage protocol of M1 and PFC stimulation for approximately 1,800 pulses per treatment day for 12–24 days, for a maximum dosage of 43,200 pulses. The effects on motor symptoms (as measured by the motor subscale of the UPDRS) were significant in four of the six studies, with three studies demonstrating that the largest effects were in individuals with advanced disease. Furthermore, the double-blind, sham-controlled trial by Spagnolo et al. (2020) demonstrated that active Deep TMS significantly improved outcomes on the UPDRS tremor subscale. From a safety perspective, AE rates were very low and consistent with the AE rates of the H4 Coil in previously published multisite randomized clinical trials (Tendler et al., 2023). The efficacy of Deep TMS in PD is likely to be dependent on patient selection. These data suggest that individuals with advanced PD may experience the greatest benefit – including a clinically significant reduction on the UPDRS following 12 treatments of the two-stage TMS treatment protocol. Deep TMS for PD may increase “on time” for these patients independent from an increase in their levodopa dose. Studies 1, 3, 4, and 6 indicate that more severe patients (UPDRS score >25 at baseline and disease duration >5 years) are more likely to respond well to TMS treatment.

In addition to motor symptoms, the two-stage Deep TMS protocol appears to have a promising impact on non-motor activities of daily living and mood symptoms of PD. Deep TMS benefits can last for several months after the treatment. At this point it is possible that further follow-up maintenance treatments may have therapeutic benefit. The best data available currently is from Cohen et al. (2018) who did one session per day for weeks 9–12. There has also been a case series with the H1 Coil (Tendler et al., 2014). Further studies are also required to determine the optimal dose of Deep TMS and the durability of Deep TMS effects on motor and non-motor symptoms in these patients. Transient dyskinesias during stimulation suggest that there may be an upper limit of tolerability.

### What is the biological rationale for a two-stage H-Coil TMS protocol?

Although the mechanism through which this two-stage H-Coil TMS protocol improves motor and non-motor symptoms in these advanced PD patients is not clear, the influence of TMS on dopamine and the ability of the H-Coils to target the PFC and motor cortex bilaterally likely play a large role. The neurodegeneration and functional disruption in PD is both anatomically and functionally diffuse, affecting multiple cortico-basal-ganglia circuits (Perovnik et al., 2023). PD is typically thought to result from a loss of dopamine producing cells in substantia nigra and an imbalance in the direct and indirect basal ganglia pathways.

The largest study in this report was a double-blind sham-controlled clinical trial in 60 patients that utilized 10 Hz stimulation to both M1 and the PFC. This study (Spagnolo et al., 2020) found a significant effect on motor subscale of the UPDRS bilaterally. They also had a significant effect on tremor. The efficacy of this protocol may be based on the observation that 10 Hz TMS can increase subcortical dopamine. A set of positron emission tomography studies by Strafella et al. (2001, 2003, 2005) and Strafella and Paus (2001) demonstrated that 10 Hz TMS delivered to the PFC and to the motor cortex reliably leads to a decrease in C11-raclopride binding (implying an increase in dopamine release) in the caudate and putamen in both healthy controls and PD (review: Kinney and Hanlon, 2022). Thus, it is likely that the 10 Hz TMS protocol with H-Coils is leading to a release of dopamine following both the bilateral M1 stimulation and the bilateral PFC stimulation in this two-stage protocol. This multipronged bilateral TMS approach with H-Coils may be particularly important for PD patients wherein the internal stores of dopamine in the meso-cortical (PFC) and nigrostriatal (motor) pathways are low.

In addition to these dopaminergic systems, there are several other neurotransmitter systems that have broad effects on cortical brain function, including degeneration of the cholinergic basal forebrain (Fyfe, 2018; Ray et al., 2018). These neurons project throughout the PFC and modulate glutamatergic pyramidal cells involved in attention, memory, and mood (McCormick, 1993). Degeneration of the forebrain appears to have significant implications for non-motor PD symptoms. For these reasons, H-Coils that have broad electric fields which are able to stimulate the wide projection zones of forebrain neurons may in fact be beneficial to addressing the breadth of motor and non-motor symptoms experienced by many PD patients.

Regarding the frequency of TMS stimulation, this two-stage protocol relies on high frequency (10+ Hz) TMS delivered to both the PFC and M1. This is the frequency that was used to demonstrate that TMS causes dopamine release in the caudate and putamen (Strafella et al., 2001, 2003, 2005). In the past, however, many TMS studies for PD have used 1 Hz M1 stimulation. It is possible that 1 Hz either decreases dopamine release (Malik et al., 2018) or is an insufficient dose to cause a therapeutic effect. The previous focus on 1 Hz is based on paired-pulse TMS studies demonstrating PD patients have deficits in intracortical inhibition and evidence it may be restored using TMS (Di Lazzaro et al., 2010; Ni et al., 2013; Saravanamuttu et al., 2021). Determining whether 1 or 10 Hz stimulation is superior to improving motor symptoms has yet to be definitively addressed.

Given the well-known ability of high frequency prefrontal stimulation to alleviate depression, it is also possible that prefrontal stimulation directly contributes to mood improvements (Pell et al., 2022). Although less well-established, prefrontal TMS may additionally contribute to improvements through modification of cognitive processes which are disrupted in PD including impaired executive function. TMS has been shown to produce modest changes in cognition in other neurodegenerative disease populations (Leocani et al., 2020). Taken together, the mood and cognitive impact of the H-Coil protocol may have both direct and downstream effects on motor and non-motor symptom severity.

## How might this two-stage TMS protocol fit into the continuum of care for PD patients?

Given the promise of H-Coil TMS in addressing both motor and non-motor aspects of PD symptomology, it is important to consider its clinical implementation. As previously discussed, there are several well-established first and second-line treatments to address motor symptoms, including dopaminergic medication, surgical ablation, and DBS. These standard treatments have several key gaps that H-Coil TMS has potential to fill. First, we observed that across studies side effects from TMS were mild and transient. While most PD patients see benefit from their dopaminergic medications, severe side effects including dyskinesias and neuropsychiatric side effects make high doses of these medications intolerable. TMS may be used in conjunction with lower doses of dopaminergic medications, resulting in reduced side effect burden while maintaining motor improvement. These effects are likely to be especially relevant in patients that have been on dopaminergic medications for some time, or which are in the later stages of their disease progression. This is supported by our finding that multiple H-Coil TMS studies reported that effects were greatest for patients with greater disease severity and progression. Future studies are needed to see the extent to which dopaminergic medications can be reduced with the addition of TMS.

The second gap in treatment where H-Coil TMS may benefit patients is for individuals with cognitive and mood symptoms. Cognitive impairment, apathy, and depression are major barriers for PD treatment as it can reduce adherence to medication regimens, reduce effective participation in therapy and make patients ineligibility for invasive brain stimulation treatments such as DBS. Thus, the H-Coil protocol for PD is a unique addition to the current tools available to treat PD by targeting the circuitry underlying two domains which are traditionally not addressed in unison. These potential benefits to cognition contrast DBS therapy where there is some evidence that subthalamic DBS may in fact worsen executive function such as verbal fluency.

Finally, there is growing evidence that TMS may be able to address symptoms which fail to respond to dopaminergic medications and DBS (Barbe et al., 2020). The most prominent of these include axial symptoms such as freezing of gait and speech issues (Barbe et al., 2020). Several studies have demonstrated that TMS may improve freezing of gait for example (review: Kim et al., 2019). Freezing of gait may benefit particularly well from this H-Coil protocol as these patients have difficulty with dual tasking and walking under time pressure implicating motor, limbic and cognitive circuitry (Schaafsma et al., 2003). Furthermore, H-Coil TMS has a deeper field than traditional figure-8 TMS coils, allowing the lower limb portion of M1 to be reliably stimulated. While not the focus of these H-Coil studies, several figure-8 TMS studies have already shown promising results that TMS can improve freezing of gait. Targets for freezing of gait have generally included single site stimulation of the SMA, primary motor cortex and the PFC.

H-Coil TMS may be well suited to fit into an increasingly personalized and interdisciplinary treatment approach for PD. PD is highly heterogenous in its clinical presentation (Greenland et al., 2019). For example, the rate of disease progression varies between tremor predominant subtypes of PD and postural instability gait difficulty (PGID) subtypes. Genetic subtypes are increasingly being used as biomarkers for PD prognosis and treatment approach. The glucocerebrosidase gene (GBA) is associated with worse non-motor cognitive symptoms and has even been associated with poorer DBS outcomes (Pal et al., 2022). PD subtypes such as these may benefit from TMS where traditional therapeutic approaches often fall short. Thus, subtypes should be considered as a factor in future H-Coil TMS clinical trials to determine if their relationship to response.

There are a few limitations to reviewing these studies that limited interpretations. The most frequently reported outcome measure was the UPDRS; however, both the original UPDRS and the revised MDS-UPDRS versions were used across the different studies. These scores cannot be converted into one other quantitatively making direct comparisons difficult. Furthermore, subject and item responses from the UPDRS were not available, limiting conclusions about specific symptoms. Most studies had small sample sizes or were divided into multiple arms limiting statistical power. It is also important to note that of the six studies four had a control group (two sham, two active comparator), and two were open label studies. More randomized controlled studies would strengthen the validity of the observed effects and clarify the relative effectiveness versus other techniques.

Despite these limitations, Deep TMS for PD studied in 220 patients appears to have promising results over a number of regionally diverse sites including Israel, Italy, Chile, and Germany. The majority of the studies focused on the motor symptoms of PD. Additionally, two studies observed effects in non-motor symptoms as well. Given the role of the PFC in mood and cognitive control, further research in this area may provide fruitful insights in to the potential use of Deep TMS to improve the non-motor symptoms of PD which can be particularly resistant to pharmaceutical treatment. As always, this research should be done in a manner that is consistent with globally acceptable ethical principles of research requiring consent and acknowledgment that while TMS for PD has clearance in many countries, it is not yet cleared for clinical use in other countries including the United States. Before a United States clinician offers Deep TMS for PD to a patient outside of a research study, consent and clinical rationale must be documented. Rates should not exceed those of an approved TMS indication. Promotion of experimental treatments is forbidden to manufacturers by the FDA, but providers may be allowed to advertise off label services depending on local medical board regulations.

Deep TMS is an acceptable and well-tolerated approach in patients with PD, which is highlighted by the low rate of drop out and limited AEs. The rates of AEs are lower than for currently FDA indications including major depression, OCD and smoking cessation. Further investigation into Deep TMS and how it may fit into the continuum of care for PD patients is warranted based on the reviewed literature.

## Author contributions

CH: Methodology, Supervision, Visualization, Writing – review & editing. DL: Validation, Writing – review & editing. GP: Data curation, Visualization, Writing – review & editing. YR: Data curation, Methodology, Visualization, Writing – review & editing. AZ: Investigation, Writing – review & editing. AT: Conceptualization, Investigation, Project administration, Writing – original draft.
