# Supplementary material for: Bilateral deep transcranial magnetic stimulation of motor and prefrontal cortices in Parkinson’s disease: a comprehensive review
Source: Front Hum Neurosci. 2024 Jan 24;17:1336027. doi: 10.3389/fnhum.2023.1336027 (PMC10847590; doi:10.3389/fnhum.2023.1336027)
Supplement: Supplementary file 1 [file Data_Sheet_1.DOCX]

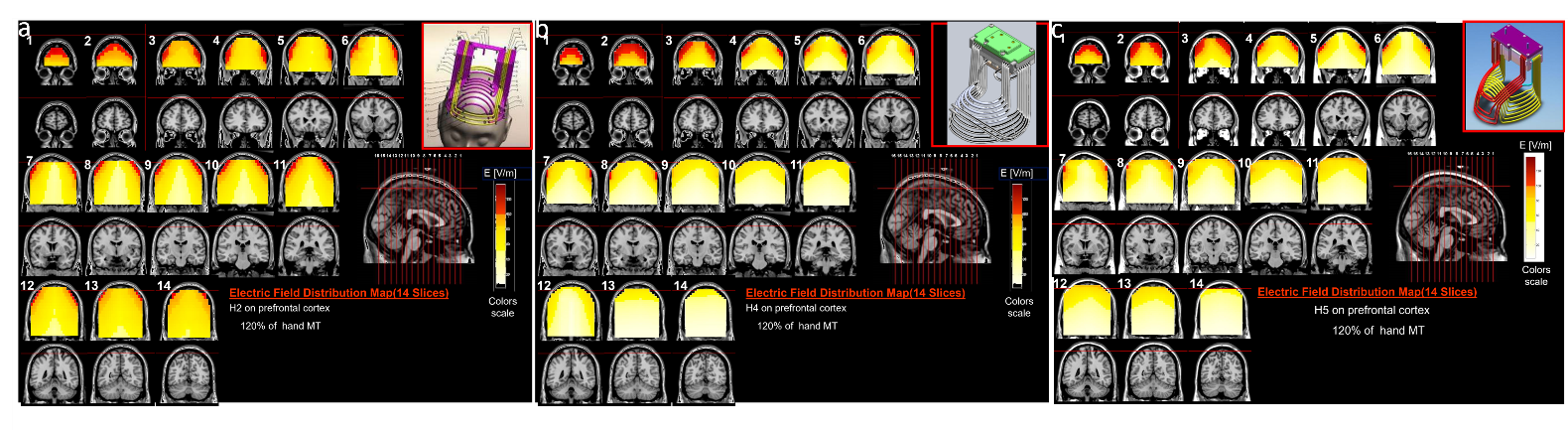


**Figure 1.** Colored field maps for the H2 (a), H4 (b) and H5 (c) coils showing electric field distribution within the brain, when located over the prefrontal cortex, indicating the electric field absolute magnitude in each pixel over 14 coronal slices 1 cm apart. The maps were adjusted to the average percentage of the maximal stimulator output required to achieve 120% of the hand rMT. The red pixels indicate field magnitude ≥ the threshold for neuronal activation, which was set to 100 V/m.


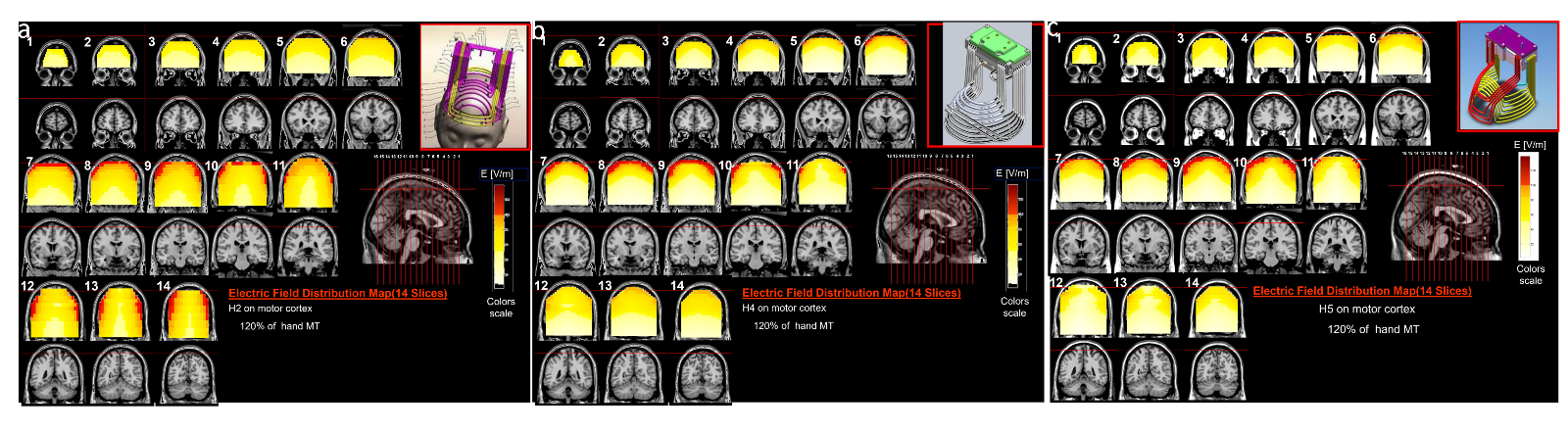


**Figure 2.** Colored field maps for the H2 (a), H4 (b) and H5 (c) coils showing electric field distribution within the brain, when located over the motor cortex, indicating the electric field absolute magnitude in each pixel over 14 coronal slices 1 cm apart. The maps were adjusted to the average percentage of the maximal stimulator output required to achieve 120% of the hand rMT. The red pixels indicate field magnitude ≥ the threshold for neuronal activation, which was set to 100 V/m. As can be seen the field distribution of the three coils is very similar, with the only exception that the H2 induces supra-threshold stimulation also at occipital cortex regions.
